# Expression of stromal cell-derived factor 1 and CXCR7 ligand receptor system in pancreatic adenocarcinoma

**DOI:** 10.1186/1477-7819-12-348

**Published:** 2014-11-18

**Authors:** Zhen Liu, Xu-Yong Teng, Xiang-Peng Meng, Bao-Sheng Wang

**Affiliations:** Department of General Surgery, Shengjing Hospital, China Medical University, No. 36 Sanhao Street, Shenyang, 110004 China

**Keywords:** chemokine, chemokine receptors, CXCR7, pancreatic neoplasms, SDF-1

## Abstract

**Background:**

Stromal cell-derived factor 1 (SDF-1) is a chemokine that is expressed in some cancer cells and is involved in tumor cell migration and metastasis. CXCR7, a novel receptor for SDF-1, has been identified recently. Research has demonstrated that SDF-1/CXCR7 interaction could play an important role in cancer progression. In this study, we aimed to investigate the expression of the SDF-1/CXCR7 ligand receptor system and the relationship between this expressions and clinicopathological characteristics in pancreatic adenocarcinoma.

**Methods:**

Expressions of SDF-1 and CXCR7 in 64 cases of pancreatic adenocarcinoma tissue and 24 cases of normal pancreatic tissue were detected immunohistochemically.

**Results:**

Expressions of SDF-1 and CXCR7 were negative in normal pancreatic tissues. Respectively, positive expression rates of SDF-1 and CXCR7 in pancreatic adenocarcinoma were 45.3% and 51.6%. The expression of SDF-1 correlated with histological grades; the expression rate in moderate to low differentiation was higher than in high differentiation (*P* <0.05). The expression of CXCR7 positively correlated with lymph node metastasis (*P* <0.05). A log-rank test showed that the expression of SDF-1^+^/CXCR7^+^ correlated with poor prognosis (*P* <0.05).

**Conclusions:**

The SDF-1/CXCR7 receptor ligand system may take part in invasive progression and metastasis of pancreatic adenocarcinoma, and might be useful as an index for evaluating invasiveness and prognosis.

## Background

Pancreatic adenocarcinoma is highly aggressive and has a poor prognosis. Despite the high mortality associated with this disease, the biology involved in the development of pancreatic adenocarcinoma remains poorly understood. Invasion and metastasis are important factors that affect the prognosis of this cancer. Stromal cell-derived factor 1 (SDF-1) is a chemotactic factor for T cells, monocytes, hematopoietic progenitor cells, dendritic cells, endothelial cells, and tumor cells [1–4], and plays a role in a number of important physiological processes including leukocyte trafficking and vasculogenesis [5, 6]. More importantly, SDF-1 plays a crucial role in the process of invasion and metastasis of tumor cells [7]; it stimulates proliferation, dissociation, migration, and invasion in a wide variety of tumor cells, including breast cancer cells, pancreatic cancer cells, and hepatocellular carcinoma cells [8, 9]. Recently, a novel receptor for SDF-1, called CXCR7, has been identified and it has been hypothesized as a new molecular link in the chain of connections between inflammation and cancer [10]. CXCR7 mediates a broad range of cellular activities, including proliferation, survival, and adhesion by binding with SDF-1 [10]. In recent years, upregulation of CXCR7 has been reported to promote lung and breast tumor growth [7] and to increase prostate cancer metastasis [11]. These results provide a reasonable basis for a proposal that the SDF-1/CXCR7 interaction could play an important role in cancer progression.

In this study, we explored the expression of SDF-1/CXCR7 receptor ligand system and the relationship between this expression and clinicopathologic characteristics in pancreatic adenocarcinoma.

## Methods

### Materials

A total of 64 patients who had undergone pancreatoduodenectomy and were diagnosed with pancreatic ductal adenocarcinoma by histologic examination were included in this study. Pancreatic adenocarcinoma samples were obtained from the Shengjing Hospital of China Medical University from 2010 to 2012. In all, 24 samples of normal pancreatic tissue acquired surgically from patients who received an operation due to trauma or benign pancreatic tumor were used in this study. All participating patients signed the informed consent. The study protocol conformed to the Declaration of Helsinki and was approved by the Ethics Committee in the Shengjing Hospital of China Medical University.

### Immunohistochemistry

Paraffin-embedded tissues were sectioned at 4 μm thickness. Antigen retrieval was performed at 95°C for 20 minutes in a sodium citrate buffer solution. Endogenous peroxidase activity was inhibited by incubation with 3% hydrogen peroxide for 10 min at room temperature. The sections were incubated in 5% goat serum for 15 min, to block any nonspecific reaction. The sections were incubated with mouse anti-human SDF-1 (R&D company, 1:100 dilution) or CXCR7 (R&D company, 1:100 dilution) at 4°C overnight. After incubation, the sections were washed in PBS for 10 min, and were incubated with goat anti-mouse IgG biotinylated second antibody (MAIXIN_BIO, China) for 1 h at room temperature and thereafter incubated in streptavidin-peroxidase complex for 30 min. Diaminobenzidine chromogen was then added to the sections. Expressions of SDF-1 and CXCR7 were quantified using a visual grading system based on the extent of staining (percentage of positive tumor cells graded on a scale from 0 to 3: 0, <5%; 1, 5 to 25%; 2, 26 to 50%; 3, >50%) and the intensity of staining (graded on a scale of 0 to 3: 0, none; 1, weak staining; 2, moderate staining; 3, strong staining). The combined extent (*E*) and intensity (*I*) of staining was obtained by calculating the product of *E* and *I* (*EI*), which varied from 0 to 6 for each spot. Negative expression was indicated for an *EI* score of 0 or 1 and positive expression for *EI* >2.

### Statistical analysis

Fisher’s exact tests were used to analyze the relationship between the expression of SDF-1 and CXCR7 and clinicopathological characteristics. Survival curves were constructed using the Kaplan-Meier method and the log-rank test was used to evaluate the statistical significance of differences. All data were analyzed using SPSS 13.0 software (SPSS Inc., Chicago, IL); *P* <0.05 was considered significant.

## Results

### Patients’ characteristics

Of the 64 pancreatic adenocarcinoma patients, the median age was 58 years (range 41 to 80 years), including 44 men and 20 women. No patients received preoperative chemotherapy or radiotherapy. All cases were accompanied by detailed clinical and surgical records. High differentiation was noted in 14 patients, and moderate to low differentiation in 50. The tumor-node-metastasis (TNM) stage was I or II in 57 cases and III or IV in 7 cases. Lymph node metastasis was observed in 37 patients. The patients’ background factors are summarized in Table 1. The follow-up time was 3 to 26 months.

**Table 1:** **Correlation between SDF-1 and CXCR7 expression and clinicopathological characteristics in pancreatic adenocarcinoma**

Clinicopathological characteristics	SDF-1 (cases)			CXCR7 (cases)		
	Positive	Negative	*P*	Positive	Negative	*P*
Sex:						
Male	18	26	>0.05	25	19	>0.05
Female	11	9		8	12	
Age:						
≤58	18	17	>0.05	20	15	>0.05
>58	11	18		13	16	
Tumor size:						
≤2 cm	3	10	>0.05	5	8	>0.05
>2 cm	26	25		8	23	
Histological grade:						
High	3	11	<0.05	8	6	>0.05
Moderate or low	26	24		25	25	
TNM stage:						
I or II	26	31	>0.05	30	27	>0.05
III or IV	3	4		3	4	
Lymph node metastasis:						
Positive	17	20	>0.05	25	12	<0.05
Negative	12	15		8	19	
Distant metastasis:						
Positive	3	3	>0.05	3	3	>0.05
Negative	26	32		30	28	

### Expression levels of SDF-1 and CXCR7 protein in pancreatic adenocarcinoma and normal pancreatic tissues

**Figure 1:**
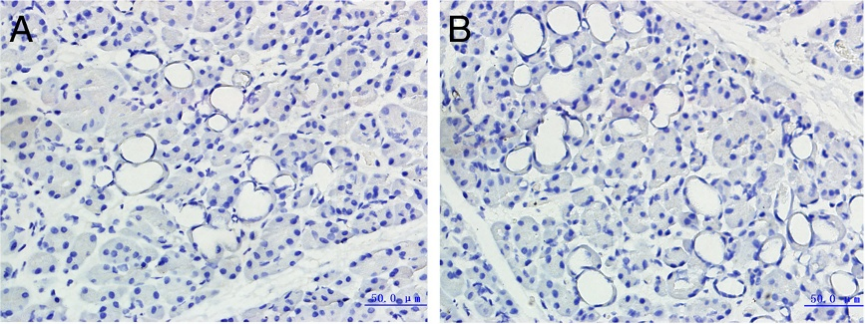
**Immunochemical staining of SDF-1 and CXCR7 in normal pancreatic tissue. (A)** Negative expression of SDF-1 (×400). **(B)** Negative expression of CXCR7 (×400).

**Figure 2:**
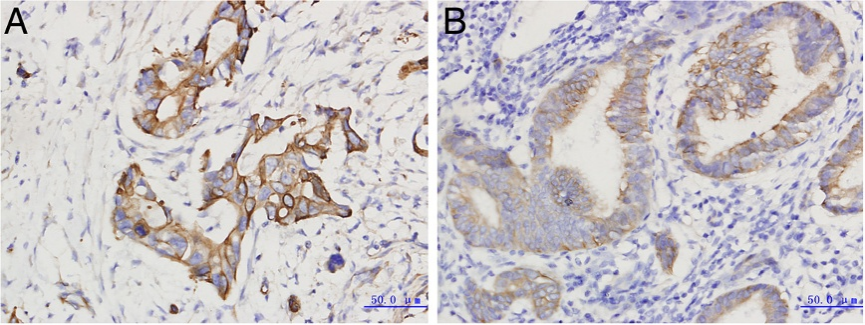
**Immunochemical staining of SDF-1 and CXCR7 expression in pancreatic adenocarcinoma tissue. (A)** Strong membranous and cytoplasmic staining for SDF-1 (×400). **(B)** Strong membranous and cytoplasmic staining for CXCR7 (×400).

In normal pancreatic tissue, SDF-1 and CXCR7 are both negative (Figure 1). In cancer tissues, SDF-1 and CXCR7 is highly expressed in ductal cells, but not in acinar and stromal tissue (Figure 2). In pancreatic adenocarcinoma, the positive expression rates of SDF-1 and CXCR7 were 45.3% (29/64) and 51.6% (33/64), respectively. The expression rates of SDF-1 and CXCR7 in cancer tissues were significantly higher than normal tissues (*P* <0.05).

### Correlation between SDF-1 and CXCR7 expressions and clinicopathological characteristics in pancreatic adenocarcinoma

We analyzed the relationship between the expressions of SDF-1 and CXCR7 and clinicopathological characteristics in pancreatic adenocarcinoma. The results showed that SDF-1 expression was not related with age, sex, size of tumor, TNM stage, lymph node metastasis, or distant metastasis (Table 1). The expression of SDF-1 correlated with histological grade of pancreatic adenocarcinoma; the expression rate of the moderate to low differentiated group was higher than that of the highly differentiated group (*P* <0.05). Expression of CXCR7 was related with lymph node metastasis, and the expression rate of CXCR7 in the group with lymph node metastasis was higher than that of the group without lymph node metastasis (*P* <0.05). There was no relationship between CXCR7 expression and age, sex, size of tumor, histological grade, TNM stage, or distant metastasis (Table 1).

### Relationship between the expressions of SDF-1 and CXCR7 and survival time

**Figure 3:**
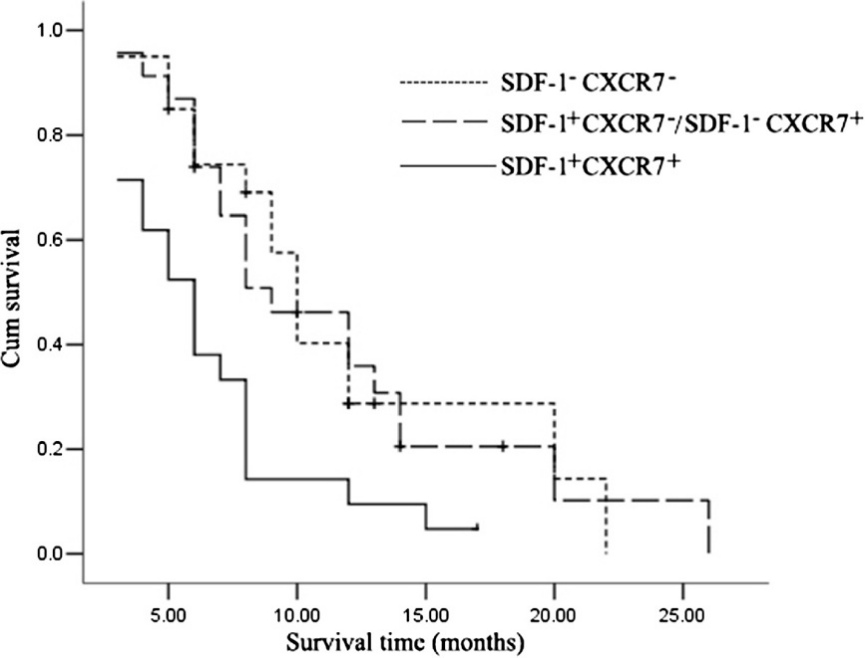
**Kaplan-Meier curves for survival in patients with pancreatic adenocarcinoma.**

Single analysis shows that there is no relation between the expression of SDF-1 and CXCR7 and prognosis. Combining analysis of the relationship between the expressions of SDF-1 and CXCR7 and prognosis reveals that the median survival time of the SDF-1^+^CXCR7^+^ group was 6 months, of the SDF-1^+^CXCR7^−^/SDF-1^−^CXCR7^+^ group was 9 months, and of the SDF-1^−^CXCR7^−^ group was 10 months. The survival time of the SDF-1^+^CXCR7^+^ group was significantly shorter than that of the SDF-1^+^CXCR7^−^/SDF-1^−^CXCR7^+^ group and the SDF-1^−^CXCR7^−^ group (*P* <0.05) (Figure 3).

## Discussion

Chemokines are a family of small cytokines with chemotaxis. In the past, chemokines were considered important regulators in the development, differentiation, and anatomic location of leukocytes [12, 13]. However, recent studies have indicated that chemokines and their receptors played a critical role in the generation and development in many types of malignant tumor, and that this role was bidirectional [14, 15]. Stromal cell-derived factor 1 is a chemokine that is expressed in some cancer cells and is involved in tumor cell migration and metastasis [16, 17]. For many years, it was believed that CXCR4 was the only receptor for SDF-1. However, several recent reports have provided evidence that CXCR7 is another receptor of SDF-1. As with SDF-1/CXCR4, the SDF-1/CXCR7 biological axis is involved in several aspects of tumorigenesis and the development and metastasis of tumors [11, 18, 19].

CXCR7 is present on the surface of many different malignant cell types [10], and on tumor-associated blood vessels, but not on normal vasculature [7]; it promotes the survival of tumor cells by preventing apoptosis, and increasing adhesion properties and dissemination, but does not mediate chemotaxis towards SDF-1 [10]. CXCR7 has been shown to induce proliferation of lung, prostatic, and breast cancer cell lines, and supported tumor growth enhancement and dissemination in a breast cancer xenograft mouse model [7, 10, 11]. CXCR7 is poorly expressed in normal somatic cells. The literature suggests that CXCR7 is highly expressed in glioma, colon cancer, lung cancer, breast cancer, prostatic cancer, and tumor-associated vessels. However, neoplastically non-transformed tissues express little CXCR7 protein; CXCR7 is only detectable at the mRNA level by Northern blotting [7, 20, 21]. In our study, SDF-1 and CXCR7 was expressed in the ductal cells of pancreatic adenocarcinoma tissue; the expression rates of SDF-1 and CXCR7 were 45.3% and 51.6%, but the expressions of SDF-1 and CXCR7 were negative in normal pancreatic tissues. The expression difference between cancer tissues and normal tissues was significant (*P* <0.05). This result suggests that the SDF-1/CXCR7 biological axis might play a role in pancreatic tumorigenesis.

We also analyzed the relationship between SDF-1/CXCR7 and the pancreatic adenocarcinoma biocharacter. The expression of SDF-1 correlated with histological grades; the expression rate in moderate to low differentiation was higher than in high differentiation (*P* <0.05). The expression of CXCR7 positively correlated with lymph node metastasis (*P* <0.05). These results suggest that expression of SDF-1 and CXCR7 might be involved in invasion and metastasis of pancreatic cancer cells. Expression of SDF-1 was high in the lymph nodes and liver [22], which were the most common destinations of pancreatic adenocarcinoma metastasis, so pancreatic cancer cells expressing CXCR7 might migrate to corresponding tissue through the expression gradient of SDF-1.

Degree of differentiation and lymph node metastasis were prognostic factors of pancreatic adenocarcinoma; low differentiation and lymph node metastasis indicated poor prognosis [23, 24]. Our research showed that the expression of SDF-1 and CXCR7 was related to histological grades and lymph node metastasis of pancreatic adenocarcinoma, which means that their expression might affect the survival of pancreatic adenocarcinoma patients. Through statistical analysis, a single analysis shows that there is no relation between expression of SDF-1 and CXCR7 and prognosis. Combining analysis of the relationship between expression of SDF-1 and CXCR7 and prognosis revealed that the median survival time of the SDF-1^+^CXCR7^+^ group was 6 months, of the SDF-1^+^CXCR7^−^/SDF-1^−^CXCR7^+^ group was 9 months, and of the SDF-1^−^CXCR7^−^ group was 10 months. The survival time of SDF-1^+^CXCR7^+^ group is significantly shorter than the SDF-1^+^CXCR7^−^/SDF-1^−^CXCR7^+^ group and the SDF-1^−^CXCR7^−^ group (*P* <0.05).

## Conclusions

Our data demonstrate that the SDF-1/CXCR7 biological axis might be involved in the invasion and metastasis of pancreatic adenocarcinoma. Both SDF-1 and CXCR7 might be useful markers for judging prognosis of pancreatic adenocarcinoma. Therefore, blocking this chemokine receptor’s pathway with a chemokine receptor antagonist or inhibitor might prove to be useful in a new strategy to prevent pancreatic adenocarcinoma development.

## Abbreviations

PBS: phosphate-buffered saline
SDF-1: stromal cell-derived factor 1
TNM: tumor-node-metastasis.

## References

[1] Bleul CC, Fuhlbrigge RC, Casasnovas JM, Aiuti A, Springer TA (1996). A highly efficacious lymphocyte chemoattractant, stromal cell-derived factor 1 (SDF-1). J Exp Med.

[2] Ara T, Nakamura Y, Egawa T, Sugiyama T, Abe K, Kishimoto T, Matsui Y, Nagasawa T (2003). Impaired colonization of the gonads by primordial germ cells in mice lacking a chemokine, stromal cell-derived factor-1 (SDF-1). Proc Natl Acad Sci USA.

[3] Askari AT, Unzek S, Popovic ZB, Goldman CK, Forudi F, Kiedrowski M, Rovner A, Ellis SG, Thomas JD, DiCorleto PE, Topol EJ, Penn MS (2003). Effect of stromal-cell-derived factor 1 on stem-cell homing and tissue regeneration in ischaemic cardiomyopathy. Lancet.

[4] Ma Q, Jones D, Borghesani PR, Nagasawa T, Kishimoto T, Bronson RT, Springer TA (1998). Impaired B-lymphopoiesis, myelopoiesis, and derailed cerebellar neuron migration in CXCR4- and SDF-1-deficient mice. Proc Natl Acad Sci USA.

[5] Aiuti A, Webb IJ, Bleul C, Springer T, Gutierrez-Ramos JC (1997). The chemokine SDF-1 is a chemoattractant for human CD34^+^ hematopoietic progenitor cells and provides a new mechanism to explain the mobilization of CD34^+^ progenitors to peripheral blood. J Exp Med.

[6] Nagasawa T, Hirota S, Tachibana K, Takakura N, Nishikawa S, Kitamura Y, Yoshida N, Kikutani H, Kishimoto T (1996). Defects of B-cell lymphopoiesis and bone-marrow myelopoiesis in mice lacking the CXC chemokine PBSF/SDF-1. Nature.

[7] Miao Z, Luker KE, Summers BC, Berahovich R, Bhojani MS, Rehemtulla A, Kleer CG, Essner JJ, Nasevicius A, Luker GD, Howard MC, Schall TJ (2007). CXCR7 (RDC1) promotes breast and lung tumor growth in vivo and is expressed on tumor-associated vasculature. Proc Natl Acad Sci USA.

[8] Marchesi F, Monti P, Leone BE, Zerbi A, Vecchi A, Piemonti L, Mantovani A, Allavena P (2004). Increased survival, proliferation, and migration in metastatic human pancreatic tumor cells expressing functional CXCR4. Cancer Res.

[9] Sutton A, Friand V, Brulé-Donneger S, Chaigneau T, Ziol M, Sainte-Catherine O, Poiré A, Saffar L, Kraemer M, Vassy J, Nahon P, Salzmann JL, Gattegno L, Charnaux N (2007). Stromal cell-derived factor-1/chemokine (C-X-C motif) ligand 12 stimulates human hepatoma cell growth, migration, and invasion. Mol Cancer Res.

[10] Burns JM, Summers BC, Wang Y, Melikian A, Berahovich R, Miao Z, Penfold ME, Sunshine MJ, Littman DR, Kuo CJ, Wei K, McMaster BE, Wright K, Howard MC, Schall TJ (2006). A novel chemokine receptor for SDF-1 and I-TAC involved in cell survival, cell adhesion, and tumor development. J Exp Med.

[11] Wang J, Shiozawa Y, Wang J, Wang Y, Jung Y, Pienta KJ, Mehra R, Loberg R, Taichman RS (2008). The role of CXCR7/RDC1 as a chemokine receptor for CXCL12/SDF-1 in prostate cancer. J Biol Chem.

[12] Rot A, von Andrian UH (2004). Chemokines in innate and adaptive host defense: basic chemokinese grammar for immune cells. Annu Rev Immunol.

[13] Luther SA, Cyster JG (2001). Chemokines as regulators of T cell differentiation. Nat Immunol.

[14] Kulbe H, Levinson NR, Balkwill F, Wilson JL (2004). The chemokine network in cancer - much more than directing cell movement. Int J Dev Biol.

[15] Vicari AP, Caux C (2002). Chemokines in cancer. Cytokine Growth Factor Rev.

[16] Geminder H, Sagi-Assif O, Goldberg L, Meshel T, Rechavi G, Witz IP, Ben-Baruch A (2001). A possible role for CXCR4 and its ligand, the CXC chemokine stromal cell-derived factor-1, in the development of bone marrow metastases in neuroblastoma. J Immunol.

[17] Kryczek I, Lange A, Mottram P, Alvarez X, Cheng P, Hogan M, Moons L, Wei S, Zou L, Machelon V, Emilie D, Terrassa M, Lackner A, Curiel TJ, Carmeliet P, Zou W (2005). CXCL12 and vascular endothelial growth factor synergistically induce neoangiogenesis in human ovarian cancers. Cancer Res.

[18] Meijer J, Ogink J, Roos E (2008). Effect of the chemokine receptor CXCR7 on proliferation of carcinoma cells *in vitro* and *in vivo*. Br J Cancer.

[19] Zheng K, Li HY, Su XL, Wang XY, Tian T, Li F, Ren GS (2010). Chemokine receptor CXCR7 regulates the invasion, angiogenesis and tumor growth of human hepatocellular carcinoma cells. J Exp Clin Cancer Res.

[20] Schutyser E, Su Y, Yu Y, Gouwy M, Zaja-Milatovic S, Van Damme J, Richmond A (2007). Hypoxia enhances CXCR4 expression in human microvascular endothelial cells and human melanoma cells. Eur Cytokine Netw.

[21] Goldmann T, DrÖmann D, Radtke J, Marwitz S, Lang DS, Schultz H, Vollmer E (2008). CXCR7 transcription in human non-small cell lung cancer and tumor-free lung tissues; possible regulation upon chemotherapy. Virchows Arch.

[22] Maréchal R, Demetter P, Nagy N, Berton A, Decaestecker C, Polus M, Closset J, Devière J, Salmon I, Van Laethem JL (2009). High expression of CXCR4 may predict poor survival in resected pancreatic adenocarcinoma. Br J Cancer.

[23] Riediger H, Keck T, Wellner U, zur Hausen A, Adam U, Hopt UT, Makowiec F (2009). The lymph node ratio is the strongest prognostic factor after resection of pancreatic cancer. J Gastrointest Surg.

[24] Fujita T, Nakagohri T, Gotohda N, Takahashi S, Konishi M, Kojima M, Kinoshita T (2010). Evaluation of the prognostic factors and significance of lymph node status in invasive ductal carcinoma of the body or tail of the pancreas. Pancreas.

